# Nucleotide polymorphisms of the maize *ZmFWL7* gene and their association with ear-related traits

**DOI:** 10.3389/fgene.2022.960529

**Published:** 2022-08-10

**Authors:** Tianyun Tao, Qianfeng Huang, Zhihao Zuo, Yue Lu, Xiaomin Su, Yang Xu, Pengcheng Li, Chenwu Xu, Zefeng Yang

**Affiliations:** ^1^ Jiangsu Key Laboratory of Crop Genetics and Physiology/Key Laboratory of Plant Functional Genomics of the Ministry of Education/Jiangsu Key Laboratory of Crop Genomics and Molecular Breeding, Agricultural College of Yangzhou University, Yangzhou, China; ^2^ Jiangsu Co-Innovation Center for Modern Production Technology of Grain Crops, Yangzhou University, Yangzhou, China

**Keywords:** maize, ear -related traits, nucleotide polymorphisms, the *ZmFWL7* gene, association analysis

## Abstract

Plant *fw2.2-like (FWL)* genes, encoding proteins harboring a placenta-specific eight domain, have been suggested to control fruit and grain size through regulating cell division, differentiation, and expansion. Here, we re-sequenced the nucleotide sequences of the maize *ZmFWL7* gene, a member of the *FWL* family, in 256 elite maize inbred lines, and the associations of nucleotide polymorphisms in this locus with 11 ear-related traits were further detected. A total of 175 variants, including 159 SNPs and 16 InDels, were identified in the *ZmFWL7* locus. Although the promoter and downstream regions showed higher nucleotide polymorphism, the coding region also possessed 61 SNPs and 6 InDels. Eleven polymorphic sites in the *ZmFWL7* locus were found to be significantly associated with eight ear-related traits. Among them, two nonsynonymous SNPs (SNP2370 and SNP2898) showed significant association with hundred kernel weight (HKW), and contributed to 7.11% and 8.62% of the phenotypic variations, respectively. In addition, the SNP2898 was associated with kernel width (KW), and contributed to 7.57% of the phenotypic variations. Notably, the elite allele T of SNP2370 was absent in teosintes and landraces, while its frequency in inbred lines was increased to 12.89%. By contrast, the frequency of the elite allele A of SNP2898 was 3.12% in teosintes, and it was raised to 12.68% and 19.92% in landraces and inbred lines, respectively. Neutral tests show that this locus wasn’t artificially chosen during the process of domestication and genetic improvement. Our results revealed that the elite allelic variants in *ZmFWL7* might possess potential for the genetic improvement of maize ear-related traits.

## Introduction

Maize (*Zea mays* L.) is a major feed, food, and fuel (ethanol) crop in many parts of the world. Although global maize production has exceeded 1.2 billion tonnes in 2021/2022 (Statista 2021), the supply is still insufficient to meet the escalating demand. Breeders’ main goal has always been to increase yield. Grain yield is primarily determined by the morphological traits of the kernel and ear. Kernel yield is a complex quantitative trait that is influenced by various ear-related traits, including ear length (EL), ear weight (EW), ear grain weight (EGW), ear row number (ERN), kernel number per row (KNR), kernel size, and kernel weight (Liu et al., 2012; Zhang et al., 2017; Zhou et al., 2020). It is known that the yield components have a higher heritability compared with the kernel yield, and selecting certain yield components may be more advantageous than directly selecting the kernel yield alone (Gupta et al., 2006; Messmer et al., 2009). Among the ear-related traits, kernel size is a major factor that determines kernel weight, which is generally influenced by kernel length, width, and thickness (Li et al., 2013). Kernel weight has become a key factor affecting yield during breeding because of the fact that kernel weight reduction cannot be compensated for by other yield factors. In addition, ear-related traits, such as ER, ERN, and KNR, also have a significant impact on maize yield (Jia et al., 2020; Wang et al., 2021). Therefore, identifying the genes associated with ear-related traits and exploiting superior allelic variation may contribute to improvements in maize high-yield breeding.

*Fruit-weight 2.2 (fw2.2),* initially isolated from the tomato (*Solanum lycopersicum* L.), is a major gene that regulates fruit size and yield (Alpert et al., 1995). This gene represents a major quantitative trait locus that is responsible for around 30% of the difference in fruit size between wild and cultivated tomatoes and functions by negatively controlling cell division during fruit development (Frary et al., 2000; Nesbitt and Tanksley, 2001; Beauchet et al., 2021). Homologs of *FWL* genes encode proteins that contain a conserved cysteine-rich PLAC8 domain (CCXXXXCPC or CLXXXXCP), and are associated with various phenotypic traits, including organ size, plant architecture, and fruit weight (Guo and Simmons, 2011; Monforte et al., 2014). Recent studies have demonstrated that *fw2.2* is transcribed specifically in the epidermis and sub-epidermis of the tomato fruit and produces new cells by regulating the division of transverse and longitudinal anticlinal cells, which promotes fruit growth (Renaudin et al., 2017; Shinozaki et al., 2018). Notably, *fw2.2* homologs are also connected with organ size or cell division in rice (*TGW2/OsCNR1*; Ruan et al., 2020), cherry (*CNR12* and *CNR20*; De Franceschi et al., 2013) and husk tomato (*CNR1*; Li and He, 2015); thus, they are also known as *Cell Number Regulator (CNR)* genes. *Fw2.2* and its *FWL/CNR* homologs can not only regulate organ size and regulate cell division, but also endow plants with a response to rhizobia inoculation (Qiao et al., 2017), cadmium (Cd) resistance (Xiong et al., 2018; Wang et al., 2019), zinc (Zn), and manganese (Mn) tolerance (Qiao et al., 2019), and Ca^2+^ signal transduction (Rosa et al., 2017).

*Fw2.2* and its *FWL/CNR* homologs belong to a complex multigene family. Thirteen *FWL/CNR* gene families have been identified in the 10 chromosomes of the maize genome. Of these, *CNR1*, is involved in a plant-specific cell proliferation mechanism and thus affects plant and fruit weight (Guo et al., 2010), whereas *CNR2* expression has been shown to be adversely associated with tissue growth activity and hybrid seedling vigor (Guo et al., 2010; Guo and Simmons, 2011). Although *FWL/CNR* plays an important role in many biological processes, studies focusing on the genetic variants of *FWL/CNR* and their association with plant phenotypic traits in maize are scarce. In the present study, we identified the *ZmFWL7* gene locus (GRMZM2G173742) from 256 inbred lines, 71 landraces, and 32 teosintes. We examined the relationship between this natural variation in this gene and several phenotypic features, including kernel yield and size. This study was designed to: 1) identify natural variations in *ZmFWL7* connected to ear-related traits; 2) determine favourable alleles which are beneficial for HKW within *ZmFWL7*; and 3) assess the significance of *ZmFWL7* in maize domestication and yield improvement.

## Materials and methods

### Plant materials, experimental design, and analysis of phenotypic data

In total, 256 elite inbred lines, 71 landraces, and 32 teosintes were collected in our study (Supplementary Table S1). All these inbred lines were planted in field in a randomized block design across three environments: the city of Sanya in Hainan province in 2015 and 2016, and the city of Yangzhou in Jiangsu province in 2017. Each line was planted with 13 plants in a single row, which made a plot 3 m long and 0.5 m wide. After harvesting and drying, three well-developed ears were used for measuring ear-related traits, including ear length (EL), ear diameter (ED), ear row number (ERN), kernel number per row (KNR), core diameter (CD), ear weight (EW), kernel length (KL), kernel width (KW), kernel thickness (KT), hundred kernel weight (HKW), and ear grain weight (EGW). Descriptive statistical analysis and ANOVA analysis of these phenotypic observations were performed using the “aov” function in R software. The best linear unbiased prediction (BLUP) values for each trait was calculated using the mixed linear model in the R package “lme4” (Bates et al., 2015). The parameters of the correlation coefficient were calculated using the R package psych.

### DNA extraction and *ZmFWL7* resequencing

Fresh leaves of 256 elite inbred lines were used to extract their genomic DNA using the modified cetyl trimethyl ammonium bromide (CTAB) method. Subsequently, the genomic sequences of the maize *ZmFWL7* locus were re-sequenced using targeted sequence capture technology on the NimbleGen platform by BGI (Beijing Genomics Institute) Life Tech Co., China. The genomic sequences and positions of the *ZmFWL7* (GRMZM2G173742) gene in the inbred line B73 (Schnable et al., 2009) were used as the reference for target capture sequencing. In order to estimate the neutral evolution of this locus, the sequences of 71 landraces and 32 teosintes were also captured using this method (Choi et al., 2009). The original re-sequencing data can be found in Supplementary Material S1.

### Analysis of genotypic data

Multiple sequence alignment of the maize *ZmFWL7* locus was performed using ClustalX software. The alignments were further manually edited to modify the putatively false alignment pairs (Larkin et al., 2007). The single nucleotide polymorphisms (SNPs), genetic and haplotype diversities of all tested lines were detected by DNASP5.0 software (Librado and Rozas, 2009). The nucleotide diversity (*π*) of the *ZmFWL7* locus was estimated as the mean number of nucleotide differences per site between sequences using the R package PopGenome. A sliding window method was used with a window size of 200 base pairs (bp) and a step length of 50 bp (Pfeifer et al., 2014).

### Marker-trait association analysis in inbred lines

TASSEL5.0 software was used to perform association analysis between the polymorphic sites of *ZmFWL7* and the BLUP values of yield-related traits collected from the 256 inbred lines (Bradbury et al., 2007). The model of mixed linear was employed for association mapping. The kinship and population structure of the population, estimated using the principal component analysis (PCA) method, were used to decrease false-positive associations. Principal component analysis (PCA) and kinship were calculated using TASSEL5.0. A significant association was defined as polymorphic with *p* < 0.01. Linkage disequilibrium (LD) was estimated between any pairs of polymorphic sites in the sequenced region of *ZmFWL7*. The LD heatmap and *r* ^*2*^ were generated using the R packages LDheatmap and pegas, respectively (Shin et al., 2006).

## Results

### Sequence polymorphisms in *ZmFWL7*

**FIGURE 1:**
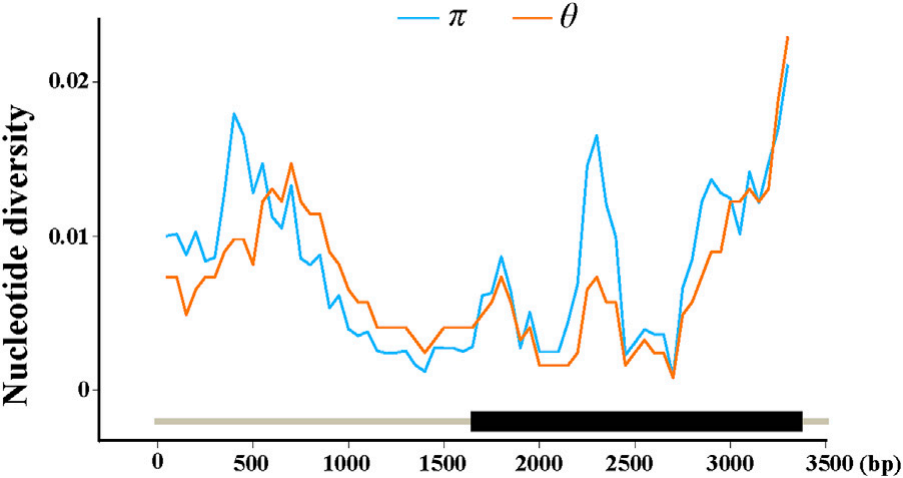
Nucleotide diversity (*π* and *θ*) estimated along the sequences of maize*ZmFWL7. π* and *θ* were calculated using the method of sliding windows of 200 bp with a step of 50 bp. The gene structure was indexed at the bottom of the coordinates.

To detect nucleotide polymorphisms in *ZmFWL7*, the full-length sequence of this locus was re-sequenced in 256 inbred lines. A total of 3,450 bp sequences were recovered using a multiple sequence alignment, comprising the 1,572 bp upstream region, the 1,734 bp coding region and 144 bp of the downstream region. Nucleotide substitution, insertion, and deletion (Indel) variations within the *ZmFWL7* locus are summarised in Table 1. In the genomic area surrounding *ZmFWL7*, a total of 175 variants, including 159 SNPs and 16 InDels, were discovered. SNPs and InDels were found every 21.7 and 251.6 kb on average. The overall nucleotide diversity (*π*) of the *ZmFWL7* locus was 0.0087 for all 256 inbred lines; however, the coding region exhibited a much lower frequency of nucleotide polymorphisms compared with the downstream region. In addition, a sliding window of 200 bp with a step length of 50 bp was used to calculate *π* and *θ* (Figure 1). The 59–144 bp area in downstream region showed the highest nucleotide diversity, with *π* = 0.0285. The 746–845 bp area in the coding region exhibited the most nucleotide diversity, with *π* = 0.0242.

**TABLE 1:** Summary of parameters for the analysis of nucleotide polymorphisms of *ZmFWL7* in inbred lines.

Parameters	Upstream	Coding region	Downstream	Full-length
Total length of amplicons (bp)	1,572	1734	144	3,450
Number of all of the sequence variants	84	67	24	175
Frequency of all of the sequence variants	0.053	0.039	0.167	0.051
Number of polymorphic sites	75	61	23	159
Frequency of polymorphic sites per bp	0.048	0.035	0.160	0.046
Number of InDels sites	19	66	4	89
Number of InDels events	9	6	1	16
Average InDel length	2.111	11.500	4.000	5.750
Frequency of InDels per bp	0.006	0.003	0.007	0.005
*π* × 1,000	8.150	7.900	24.840	8.730
*θ* × 1,000	8.100	5.980	26.840	7.830
Tajima’s *D*	0.020	0.954	−0.199	0.355
Fu and Li’s *D*	1.800**	1.744**	0.376	1.873**
Fu and Li’s *F*	1.161	1.663	0.171	1.339

*Indicates a statistical significance at *p* < 0.05 level.

** Indicates a statistical significance at *p* < 0.01 level.

### Nucleotide diversity and selection of *ZmFWL7* in inbred lines, landraces, and teosintes

**FIGURE 2:**
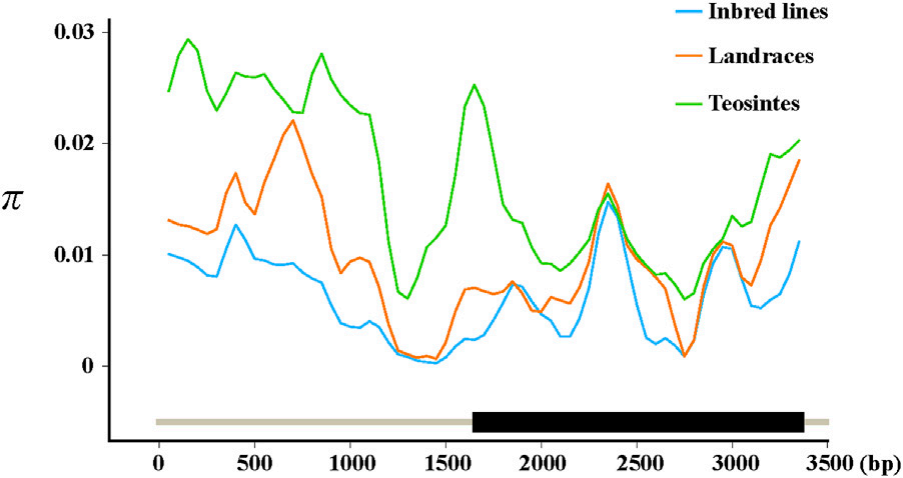
Nucleotide diversity in inbred lines, landraces, and teosintes. *π* was calculated using the sliding window method with a window size of 200 bp and a step length of 50 bp. Three lines were fitted by locally weighted regression (Loess) in R software. The parameter controlling the degree of smoothing was set as 0.1.

To see whether artificial selection played a role in the domestication of *ZmFWL7*, we compared the genetic diversity of this gene and its flanking areas in inbred lines, landraces, and teosintes (Table 2). Compared with teosintes, inbred lines and landraces exhibited lower genetic diversity in the *ZmFWL7* coding region, implying that selection occurred along the entire length of the gene sequence. In addition, we observed that the highest divergence between inbred lines and teosintes occurred in the upstream and downstream regions, whereas the coding regions were associated with a low divergence (Figure 2). These results indicate that this uneven distribution of polymorphisms may result from a lower frequency of variants in the coding region of *ZmFWL7*.

**TABLE 2:** The estimated parameters of nucleotide diversity, Tajima’*D*, Fu and Li’s *D*, and Fu and Li’s *F* of *ZmFWL7*.

Region	Parameters	Inbreds	Landraces	Teosintes	All-lines
Upstream	*π* × 1,000	8.150	14.07	28.14	12.37
	*θ* × 1,000	8.100	19.19	47.03	35.82
	Tajima’s *D*	0.020	−0.919	−1.545	−2.007*
	Fu and Li’s *D*	1.800**	−2	−1.797	−6.641**
	Fu and Li’s *F*	1.161	−1.876	−2.029	−4.927**
Coding Region	*π* × 1,000	7.900	11.12	19.09	9.74
	*θ* × 1,000	5.980	15.34	24.94	20.43
	Tajima’s *D*	0.954	−0.94	−0.896	−1.587
	Fu and Li’s *D*	1.744**	−2.871*	−1.43	−6.689**
	Fu and Li’s *F*	1.663	−2.516*	−1.48	−4.798**
Downstream	*π* × 1,000	24.840	45.4	49.42	40.38
	*θ* × 1,000	26.840	53.80	73.36	53.45
	Tajima’s *D*	−0.199	−0.488	−1.152	−0.654
	Fu and Li’s *D*	0.376	0.153	−0.855	−1.106
	Fu and Li’s *F*	0.171	−0.1	−1.122	−1.105
Full length	*π* × 1,000	8.730	13.651	24.13	11.87
	*θ* × 1,000	7.830	18.44	36.51	28.70
	Tajima’s *D*	0.355	−0.905	−1.309	−1.809*
	Fu and Li’s *D*	1.873**	−2.244	−1.643	−6.849**
	Fu and Li’s *F*	1.339	−2.036	−1.814	−4.844**

*Indicates a statistical significance at *p* < 0.05 level.

** Indicates a statistical significance at *p* < 0.01 level.

To further investigate the role of *ZmFWL7* in the domestication and refinement of maize, *ZmFWL7* sequences from three populations were examined by a neutral test, including Tajima’s *D*, and Fu and Li’s D* and F*. All of the estimation values from Tajima’s *D* in the teosintes and landraces were negative, suggesting an excess of low-frequency alleles in the landraces and teosintes. For the three populations, the Tajima’s *D* values did not reach a statistically significant level. Additionally, we found that the values for Tajima’s *D* in the inbred lines were positive, except for the downstream region, while Fu and Li’s D* estimations for this gene were significantly higher than zero, suggesting that moderate frequency alleles are present in this population.

### Phenotypic variations and association analysis

**FIGURE 3:**
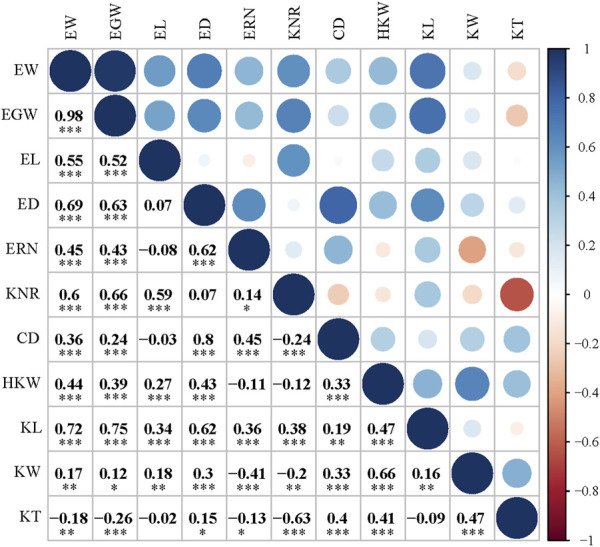
Pearson correlation coefficients for the BLUP value of 11 ear traits in 256 maize inbred lines. Abbreviations for traits are as follows: CD, core diameter; ED, ear diameter; EGW, ear grain weight; EL, ear length; ERN, ear row number; EW, ear weight; HKW, hundred kernel weight; KNR, kernel number per row; KW, kernel width. * indicates a statistical significance at *p* < 0.05 level, ** indicates a statistical significance at *p* < 0.01 level, *** indicates statistical significance at *p* < 0.001 level.

Eleven ear-related traits were determined, and the descriptive statistics are presented in Supplementary Table S2. All of these ear-related features exhibited significant variations across inbred lines in ANOVA analysis, indicating that this population has genetic characteristics for association analyses. In addition, a significant phenotypic variation in all of these traits was observed in different environments and genotype-environment interactions. It is worth noting that the block effect significantly affected all traits except CD and KT (Supplementary Table S2). Therefore, to reduce the influence of environmental effects and block effects on genetic assessment and to obtain individual stability in genetic phenotypes, the BLUP model was used for analyzing the phenotypic data. A pairwise correlation analysis was performed to evaluate the association between these phenotypic traits, and Pearson correlation coefficients (*r*) between any two phenotypic traits were determined. Interestingly, most traits were statistically significantly correlated, with EW/EGW exhibiting the greatest correlation (*r* = 0.98). Only 8 of the 55 pairwise correlation tests for ear-related traits did not reach a significant level (EL/ED, EL/ERN, EL/CD, EL/KT, ED/KNR, ERN/HKW, KNR/HKW, and KL/KT) (Figure 3). Notably, HKW showed a significant positive correlation with KL, KW, and KT, of which KW exhibited the highest correlation with HKW (*r* = 0.98, *p* < 0.001). Therefore, the study of kernel length, width, and thickness should be conducted in the context of kernel weight.

**FIGURE 4:**
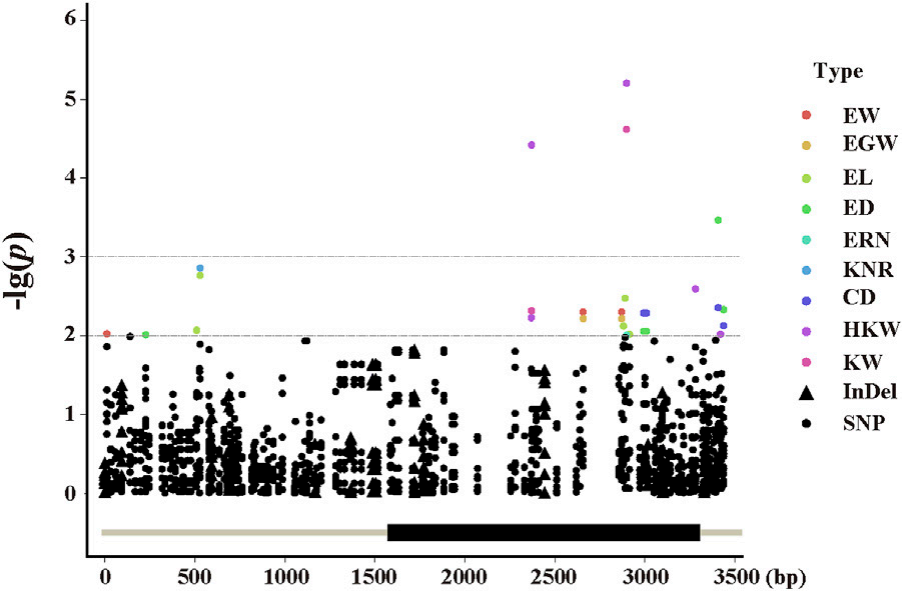
Manhattan plot by using the MLM (PCA + Kinship) model. Dots and triangles represent SNPs and Indels, respectively. Abbreviations for traits are as follows: CD, core diameter; ED, ear diameter; EGW, ear grain weight; EL, ear length; ERN, ear row number; EW, ear weight; HKW, hundred kernel weight; KNR, kernel number per row; KW, kernel width.

**FIGURE 5:**
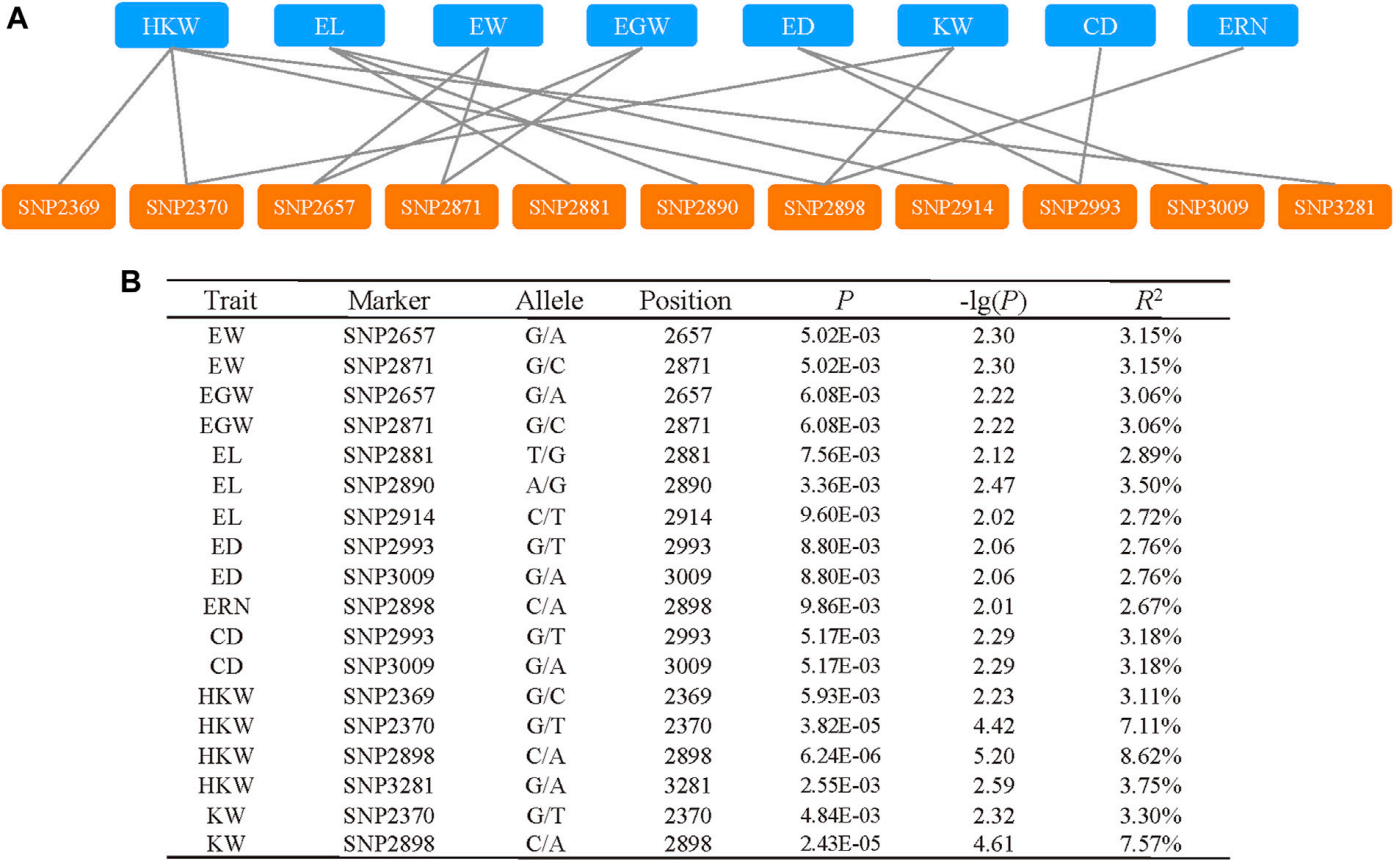
Significant markers associated with phenotypic traits. **(A)** The network between pleiotropic site and associated traits. **(B)** The information of significant markers which associated with phenotypic.

To find significant variants related with phenotypic traits, a statistical analysis of 128 variants was performed, comprising 101 SNPs and 27 InDels with a minor allele frequency (MAF) ≥ 0.01. A marker-trait association study was carried out using mixed linear models (PCA + Kinship) (Figure 4). The quantile quantile (QQ) plot indicated that the PCA and kinship were well controlled for association study in the mixed linear models (Supplementary Figure S1). And the results indicated that 11 pleiotropic sites were significantly associated, including EW, EGW, EL, ED, ERN, CD, HKW, and KW (*p* < 0.01, Figure 5A). We further analysed two pleiotropic sites (SNP2370 and SNP2898) in the coding region in which the *p*-value was less than 0.001. SNP2370 (serine to alanine) and SNP2898 (lysine to glutamine) were significantly associated with HKW, respectively, which explains the 7.11% and 8.62% phenotypic variation (Figure 5B). Moreover, SNP2898 was also significantly associated with KW, which could explain the phenotypic variation rate of 7.57%. These results demonstrate that *ZmFWL7* may be associated with HKW and KW.

**FIGURE 6:**
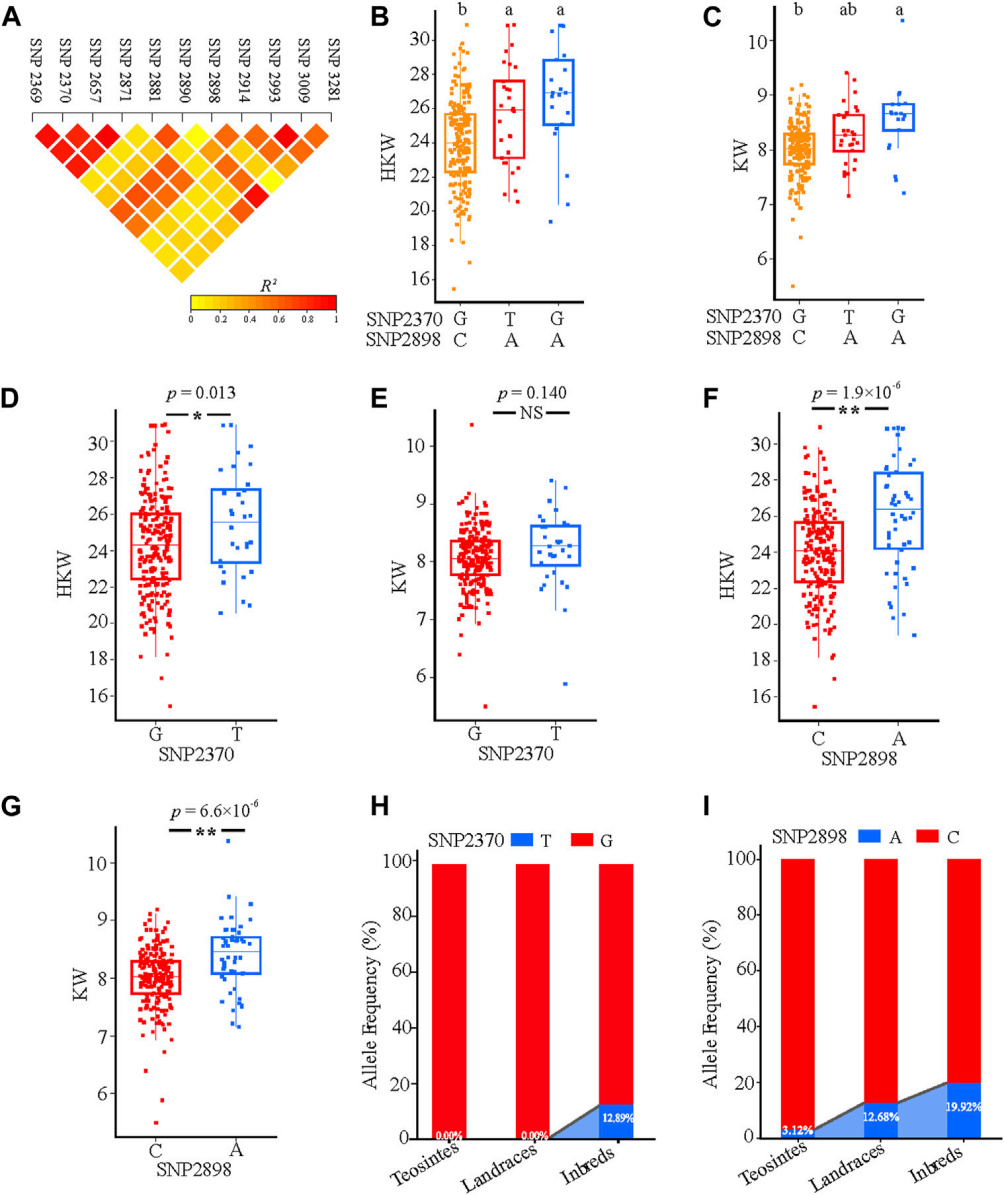
Natural variations in *ZmFWL7* were significantly associated with hundred kernel weight (HKW) and kernel width (KW). **(A)** Linkage disequilibrium (LD) heatmap for eleven significant variants associated with ear traits. **(B,C)** Haplotypes of *ZmFWL7* among natural variations based on two nonsynonymous mutation SNP2370 and SNP2898. Different letters indicate significant difference (Tukey’s HSD test at *p* < 0.05). **(D–G)** Comparison of hundred kernel weight (HKW) and kernel width (KW) between different alleles of SNP2370 and SNP2898. **(H,I)** The allele frequencies of SNP2370 and SNP2898 in inbred lines, landraces, and teosintes. *p* value for the *t* test comparing two groups carrying different alleles were indexed on the top (**p < 0.01; *p < 0.05).

Linkage disequilibrium (LD) analysis indicated that SNP2369, SNP2370, SNP2657, and SNP2871 showed a relatively high linkage with SNP2898 (Figure 6A). Among them, SNP2370 and SNP2898 showed a relatively high LD, which was associated with HKW and KW. Three haplotypes were divided based on these two SNPs, hap2, and hap3 have a significantly higher HKW than hap1. While Hap3 has a significantly higher KW than hap1 (Figures 6B,C). For SNP2370, the allele T group had a significantly higher HKW than the allele G group (Figures 6D,E). In addition, we noticed that the allele A group also contained significantly higher values for HKW and KW compared with allele C in SNP2898 (Figures 6F,G). These results indicate that allele A of SNP2898 increased maize KW and thus resulted in the HKW variant. Furthermore, we analysed SNP2370 and SNP2898 allele frequencies in three separate groups. According to the findings, the frequency of SNP2898A in teosintes was 3.12%, while the frequency increased to 12.68% and 19.92% in landraces and inbred lines, respectively. By contrast, the elite allele T of SNP2370 was absent in teosintes and landraces, while its frequency was increased to 12.89% in inbred lines (Figures 6H,I). Based on these results, we conclude that allele A of SNP2898 and allele T of SNP2370 have a positive effect on the increase of HKW in maize.

## Discussion

Candidate gene association mapping is widely employed to detect functional SNPs or alleles linked to gene-related agronomic parameters in maize, such as *Dwarf8* (Thornsberry et al., 2001) for flowering period; *ZmPGP1* (Li et al., 2019b), *GS3* (Li et al., 2010b) and *GW2* (Li et al., 2010a) for kernel shape and weight; *Zmisa2* (Yang et al., 2014) and *ZmBT1* (Xu et al., 2014) for starch properties; *ZmHKT1* (Li et al., 2019a) and *ZmMADS60* (Li et al., 2020) for root morphology; and *ZmTD1* (Liu et al., 2019). Through the method of the candidate gene association analysis, we seek to uncover the link between natural sequence variation of the maize gene *ZmFWL7* and variation of ear-related variables such as grain weight and grain width in this study. We found that a total of 11 variants in the maize *ZmFWL7* gene show associations with multiple ear-related traits, including EW, EGW, EL, ED, ERN, CD, HKW, and KW. The *ZmFWL7* gene was found to be significantly associated with kernel weight and KW, and the excellent haplotypes significantly increased HKW and KW. Endosperm filling, kernel weight and kernel size have become key parameters for maize yield, and studies have shown that kernel weight is closely related to KW, which is regulated by cell division and cell expansion (Li et al., 2018). Endosperm growth determines the majority of maize kernel size, and cell expansion runs through the whole process of endosperm development (Ji et al., 2022). Studies have shown that the *FWL* gene promotes cell division, produces new cells, and increases fruit size by regulating the division of transverse and longitudinal anticlinal cells (Renaudin et al., 2017; Shinozaki et al., 2018). In addition, the maize *CNR1* gene, a member of *FWL* gene family, increases the grain width through a plant-specific cell proliferation function (Guo et al., 2010). In this analysis, two nonsynonymous SNPs (SNP2370 and SNP2898) showed a significant association with HKW and KW simultaneously. Further analyses suggested that allele A of the SNP2898 variants significantly increased KW and HKW. The results of a phenotypic correlation analysis also revealed that the most relevant trait to HKW was KW. Therefore, we speculated that allele A of SNP2898 may increase HKW through positive regulation of KW. In addition, allele T of the SNP2370 variants significantly increased HKW. Haplotype analysis based on these two nonsynonymous SNPs also showed that hap2 with SNP2370-T and SNP 2898-A had higher HKW than hap1 with SNP2370-G and SNP 2898-C. These results showed that both SNP2370 and SNP2898 can be used as the target sites to regulate kernel weight, and therefore has important application value.

Although the selection strategies for crop genes vary at different selection stages (domestication or improvement), it is undeniable that many genes that control important agronomic traits have been subject to crop domestication and subsequent selective breeding (Liu et al., 2021). The functions and underlying mechanisms of these genes have been extensively studied and utilised (Doebley et al., 2006). The domesticated or enhanced genes in maize, wheat, and rice include a variety of biological traits, such as grain filling (*GIF1*; Wang et al., 2008, *ZmSWEET4c*; Sosso et al., 2015), flowering time (*ZmCOL3*; Jin et al., 2018), grain quality (*Wx*; Huang et al., 2020), and crop yield (*KNR2*; Chen et al., 2022). The main objectives include easy cultivation, high yield, and rich nutrition. *FWL/CNR* gene family plays a crucial function in plant development regulation (Thibivilliers et al., 2020). In this study, we detected sequence polymorphisms in *ZmFWL7* from 256 inbred lines, which yielded approximately one SNP per 35 bp in the coding region, indicating that this population has rich genetic diversity. The sequences of *ZmFWL7* in three populations were tested by the neutral evolution test. The results indicated that all of the Tajima’s *D* values for the three separate groups did not reach a statistically significant level, indicating that the *ZmFWL7* locus did not escape from neutral evolution. Of note, the allele A of SNP2898 was rare in the teosintes (3.12%), but its frequency trend increased and was 4 times higher in landraces and more than 6 times higher in inbred lines. In addition, the allele T of SNP2370 was completely absent in teosintes and landraces, but the frequency of this allele was increased to 12.89% in inbred lines. These findings indicated that these excellent allele variations have the potential values in maize breeding.

In summary, it is possible to increase kernel yield by using this gene in the maize population. Although the functional characteristics of *ZmFWL7* need to be further investigated, our findings indicate that SNP2370 and SNP2898 may be used for marker-assisted selection to improve maize breeding.

## Data Availability

The original contributions presented in the study are included in the article/Supplementary Material, further inquiries can be directed to the corresponding authors.
