# Supplementary figures and images for: Nucleotide polymorphisms of the maize ZmFWL7 gene and their association with ear-related traits
Source: Front Genet. 2022 Aug 10;13:960529. doi: 10.3389/fgene.2022.960529 (PMC9399371; doi:10.3389/fgene.2022.960529)

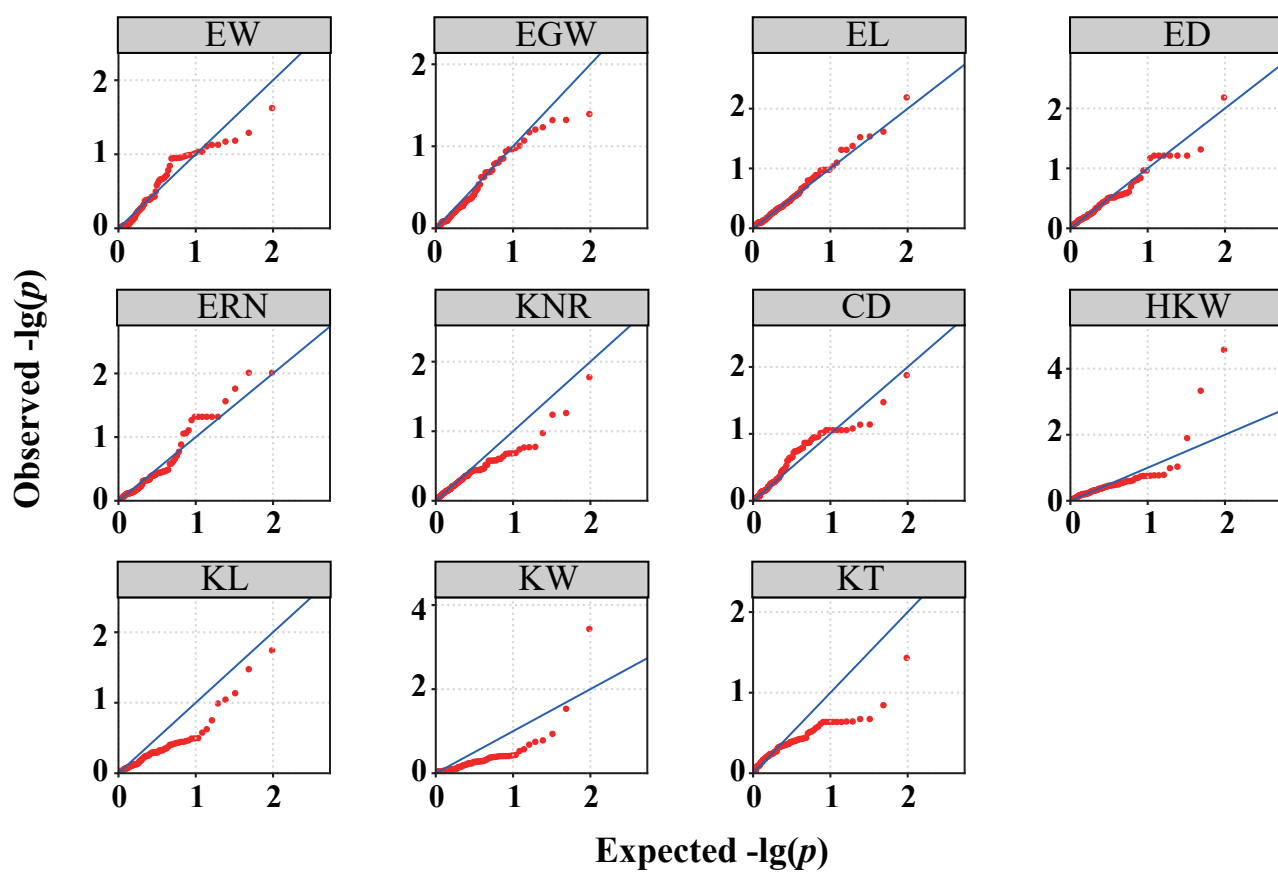

**Supplementary Figure S1.** QQ plot for the association analysis.

Supplement: Supplementary file 1 [file DataSheet7.PDF]
