# Supplementary material for: Nucleotide polymorphisms of the maize ZmFWL7 gene and their association with ear-related traits
Source: Front Genet. 2022 Aug 10;13:960529. doi: 10.3389/fgene.2022.960529 (PMC9399371; doi:10.3389/fgene.2022.960529)
Supplement: Supplementary file 3 [file DataSheet9.docx]

Supplementary Material

# Supplementary Table S1. The list of 256 inbred lines, 71 landraces and 32 teosintes used in this study.

| Category | Line ID | Lines Name | Group |
| --- | --- | --- | --- |
| Inbred line | A002 | Chang7-2 | Tang SPT |
| Inbred line | A003 | LX9801 | Tang SPT |
| Inbred line | A004 | 107 | Tang SPT |
| Inbred line | A005 | Huang518 | Tang SPT |
| Inbred line | A006 | K12 | Tang SPT |
| Inbred line | A007 | H21 | Tang SPT |
| Inbred line | A008 | Ji853 | Tang SPT |
| Inbred line | A010 | Huangzaosi | Tang SPT |
| Inbred line | A014 | QZ01 | Tang SPT |
| Inbred line | A015 | Y53 | Tang SPT |
| Inbred line | A016 | Dan598 | Lvdahonggu |
| Inbred line | A017 | Zong3-1 | Lvdahonggu |
| Inbred line | A018 | E28 | Lvdahonggu |
| Inbred line | A021 | S122 | Lvdahonggu |
| Inbred line | A024 | Dan99 | Lvdahonggu |
| Inbred line | A025 | Nx335 | Lancaster |
| Inbred line | A027 | 4CV | Lancaster |
| Inbred line | A029 | OH43 | Lancaster |
| Inbred line | A031 | BJ-4 | Lancaster |
| Inbred line | A032 | BEM | Lancaster |
| Inbred line | A034 | 8112 | Reid |
| Inbred line | A035 | K8112 | Reid |
| Inbred line | A037 | 4866 | Reid |
| Inbred line | A038 | 3189 | Reid |
| Inbred line | A039 | Tie9206 | Reid |
| Inbred line | A040 | Benyu15 | Reid |
| Inbred line | A041 | Chun2433 | Reid |
| Inbred line | A042 | 478s | Reid |
| Inbred line | A044 | 7922 | Reid |
| Inbred line | A046 | JB | Reid |
| Inbred line | A047 | S80 | Pgroup |
| Inbred line | A049 | 178 | Pgroup |
| Inbred line | A052 | Xy35 | Pgroup |
| Inbred line | A055 | Dan988 | Pgroup |
| Inbred line | A056 | 319B | Pgroup |
| Inbred line | A057 | Qi319w | Pgroup |
| Inbred line | A058 | Qi318 | Pgroup |
| Inbred line | A059 | Shen137 | Pgroup |
| Inbred line | A060 | 11099 | Tropic |
| Inbred line | A063 | 11200 | Tropic |
| Inbred line | A064 | 10533-1 | Tropic |
| Inbred line | A065 | RCML15 | Tropic |
| Inbred line | A067 | FLB01 | Tropic |
| Inbred line | A070 | KWS456 | Other |
| Inbred line | A073 | JND-1 | Other |
| Inbred line | A074 | JND-2 | Other |
| Inbred line | A075 | BJ-2 | Lancaster |
| Inbred line | A076 | QF02 | Other |
| Inbred line | A078 | QDM01 | Other |
| Inbred line | A079 | M1 | Other |
| Inbred line | A081 | WT26 | Other |
| Inbred line | A082 | M1132 | Other |
| Inbred line | A084 | QKN01M | Mixed |
| Inbred line | A089 | QKN01F | Mixed |
| Inbred line | A090 | QBN48 | Mixed |
| Inbred line | A092 | QHNZ04 | Mixed |
| Inbred line | A094 | QBN3186 | Mixed |
| Inbred line | A097 | Oh43-1 | Lancaster |
| Inbred line | A098 | Oh43-2 | Lancaster |
| Inbred line | A099 | Mo113 | Lancaster |
| Inbred line | A101 | D805 | Lancaster |
| Inbred line | A103 | LH39 | Lancaster |
| Inbred line | A104 | Mo17.351 | Lancaster |
| Inbred line | A105 | Mo17.352 | Lancaster |
| Inbred line | A106 | LH53 | Lancaster |
| Inbred line | A107 | LH55.LH47 | Lancaster |
| Inbred line | A108 | Mo17 | Lancaster |
| Inbred line | A111 | Mo17s | Lancaster |
| Inbred line | A113 | 13N-207 | Pgroup |
| Inbred line | A114 | 13N-209 | Pgroup |
| Inbred line | A115 | Nongda1145 | Pgroup |
| Inbred line | A116 | T249 | Pgroup |
| Inbred line | A117 | YJ7 | Pgroup |
| Inbred line | A118 | S651 | Pgroup |
| Inbred line | A119 | CM | Pgroup |
| Inbred line | A120 | Qi319X7 | Pgroup |
| Inbred line | A121 | JS06730 | Pgroup |
| Inbred line | A122 | Zong3.T877 | Pgroup |
| Inbred line | A123 | JS045 | Pgroup |
| Inbred line | A125 | Xin19M | Reid |
| Inbred line | A126 | K22 | Reid |
| Inbred line | A128 | 478 | Reid |
| Inbred line | A129 | DH9M | Reid |
| Inbred line | A131 | H991 | Reid |
| Inbred line | A133 | ZGF | Reid |
| Inbred line | A134 | Xian3M | Reid |
| Inbred line | A141 | Zong31s | Lvdahonggu |
| Inbred line | A143 | 13N-281 | Lvdahonggu |
| Inbred line | A144 | 13N-282 | Lvdahonggu |
| Inbred line | A147 | 340D | Lvdahonggu |
| Inbred line | A151 | T75 | Lvdahonggu |
| Inbred line | A152 | 302M | Lvdahonggu |
| Inbred line | A153 | Y09 | Lvdahonggu |
| Inbred line | A156 | T2 | Mixed |
| Inbred line | A157 | JNHWF | Mixed |
| Inbred line | A158 | HNW | Mixed |
| Inbred line | A159 | WH8 | Mixed |
| Inbred line | A161 | J2F | Mixed |
| Inbred line | A162 | L1M | Mixed |
| Inbred line | A163 | 08M | Mixed |
| Inbred line | A166 | S181 | Mixed |
| Inbred line | A169 | A489 | Other |
| Inbred line | A170 | DK516M | Other |
| Inbred line | A174 | Wu314 | Tang SPT |
| Inbred line | A177 | A19 (13N-333) | Tang SPT |
| Inbred line | A179 | CJF | Tang SPT |
| Inbred line | A180 | Y85·C72 | Tang SPT |
| Inbred line | A183 | 926F | Tang SPT |
| Inbred line | A184 | Weike02F | Tang SPT |
| Inbred line | A186 | Suyu1F | Tang SPT |
| Inbred line | A187 | Yangguang98F | Tang SPT |
| Inbred line | A189 | Dayu3F | Tang SPT |
| Inbred line | A190 | YDF | Tang SPT |
| Inbred line | A191 | D108 | Tang SPT |
| Inbred line | A192 | D4990 | Tang SPT |
| Inbred line | A194 | S4 | Tropic |
| Inbred line | A202 | QCY7 | unknow |
| Inbred line | A204 | BL-1 | unknow |
| Inbred line | A205 | HNHZ4 | unknow |
| Inbred line | A208 | TN9F | unknow |
| Inbred line | A215 | Liao112 | unknow |
| Inbred line | A216 | L118 | unknow |
| Inbred line | A217 | L48 | unknow |
| Inbred line | A219 | CA47 | unknow |
| Inbred line | A220 | J4112 | unknow |
| Inbred line | A222 | Qi205 | unknow |
| Inbred line | A223 | U8112 | Reid |
| Inbred line | A225 | CAV886 | unknow |
| Inbred line | A229 | CML122 | unknow |
| Inbred line | A231 | GEMS56 | Lancaster |
| Inbred line | A232 | CIMBL139 | Mixed |
| Inbred line | A234 | H-33 | Mixed |
| Inbred line | A235 | TN9M | Mixed |
| Inbred line | A237 | DH1M | unknow |
| Inbred line | A240 | Weihexin | Mixed |
| Inbred line | A241 | Ao89E4 | Mixed |
| Inbred line | A243 | H18 | Mixed |
| Inbred line | A244 | 5003 | Mixed |
| Inbred line | A245 | Ye8112 | Mixed |
| Inbred line | A246 | Qi318s | Mixed |
| Inbred line | A247 | H23 | Mixed |
| Inbred line | A248 | HR962 | Mixed |
| Inbred line | A250 | BLY-2 | Mixed |
| Inbred line | A253 | M3 | Mixed |
| Inbred line | A254 | Dibai | Mixed |
| Inbred line | A255 | WAYA24 | Mixed |
| Inbred line | A256 | Jiu03 | Mixed |
| Inbred line | A257 | H32 | Mixed |
| Inbred line | A258 | Ao20 | Mixed |
| Inbred line | A259 | Zhonger02 | Mixed |
| Inbred line | A261 | H36 | Mixed |
| Inbred line | A262 | Y8H | Mixed |
| Inbred line | A264 | K36 | Mixed |
| Inbred line | A265 | H41 | Mixed |
| Inbred line | A266 | H45 | Mixed |
| Inbred line | A268 | Qun1-1 | Mixed |
| Inbred line | A269 | ZH0853 | Mixed |
| Inbred line | A270 | H34 | Mixed |
| Inbred line | A272 | K910G | Mixed |
| Inbred line | A274 | H26 | Mixed |
| Inbred line | A275 | Qun31 | Mixed |
| Inbred line | A276 | DH11F | Mixed |
| Inbred line | A281 | Huang7 | Mixed |
| Inbred line | A282 | Huotanghuang17 | Mixed |
| Inbred line | A287 | Y10H | Mixed |
| Inbred line | A288 | JN2 | Mixed |
| Inbred line | A289 | 488 | Mixed |
| Inbred line | A291 | H31 | Mixed |
| Inbred line | A292 | 35199 | Mixed |
| Inbred line | A295 | OAXA179 | Mixed |
| Inbred line | A296 | H27 | Mixed |
| Inbred line | A300 | X178 | Mixed |
| Inbred line | A303 | Jidan261M | Mixed |
| Inbred line | A305 | Longkang1 | Mixed |
| Inbred line | A306 | Lu65 | Mixed |
| Inbred line | A309 | FR218 | Mixed |
| Inbred line | A311 | Ziyu3 | Mixed |
| Inbred line | A314 | H24 | Mixed |
| Inbred line | A315 | J599-2 | Mixed |
| Inbred line | A316 | H35 | Mixed |
| Inbred line | A319 | H25 | Mixed |
| Inbred line | A322 | HN785 | Mixed |
| Inbred line | A325 | Han102 | Mixed |
| Inbred line | A326 | Yan103 | Mixed |
| Inbred line | A330 | Hun21 | Mixed |
| Inbred line | A331 | Wu96 | Mixed |
| Inbred line | A334 | BEM | unknow |
| Inbred line | A335 | 935 | Mixed |
| Inbred line | A336 | L01067 | unknow |
| Inbred line | A337 | T878 | unknow |
| Inbred line | A338 | T877 | unknow |
| Inbred line | A341 | Hai13 | Mixed |
| Inbred line | A344 | Hai18 | Mixed |
| Inbred line | A345 | Hai19 | Mixed |
| Inbred line | A349 | Hai23 | Mixed |
| Inbred line | A350 | Hai24 | Mixed |
| Inbred line | A351 | Hai25 | Mixed |
| Inbred line | A352 | Hai26 | Mixed |
| Inbred line | A354 | Hai29 | Mixed |
| Inbred line | A356 | TJH5F | Mixed |
| Inbred line | A357 | CY189 | Mixed |
| Inbred line | A358 | CXY | Tang SPT |
| Inbred line | A360 | XY335X | Mixed |
| Inbred line | A362 | Q1220 | Mixed |
| Inbred line | A364 | CQY7 | Mixed |
| Inbred line | A369 | QKW456-2 | Mixed |
| Inbred line | A371 | JD-1 | Mixed |
| Inbred line | A372 | JD-2 | Mixed |
| Inbred line | A373 | F8-1-11 | Mixed |
| Inbred line | A375 | M3564 | Mixed |
| Inbred line | A377 | KN-M | Mixed |
| Inbred line | A378 | DG7-1 | Mixed |
| Inbred line | A379 | LY-1 | Mixed |
| Inbred line | A383 | BJ-1 | Lancaster |
| Inbred line | A384 | LY16F | unknow |
| Inbred line | A385 | BN189 | unknow |
| Inbred line | A386 | BN386 | unknow |
| Inbred line | A389 | PH6WC | unknow |
| Inbred line | A390 | S37 | unknow |
| Inbred line | A393 | HuangC | Tang SPT |
| Inbred line | A395 | Zong31 | Lvdahonggu |
| Inbred line | A397 | Zong3 | Lvdahonggu |
| Inbred line | A398 | PH4CV | unknow |
| Inbred line | A401 | LY16Fs | unknow |
| Inbred line | A402 | 40M | unknow |
| Inbred line | A403 | 40F | unknow |
| Inbred line | A404 | 41F | unknow |
| Inbred line | A405 | MY8M | unknow |
| Inbred line | A406 | MY8F | unknow |
| Inbred line | A407 | S35M | unknow |
| Inbred line | A408 | S35F | unknow |
| Inbred line | A409 | 11HT-29 | unknow |
| Inbred line | A410 | 12HTJ | unknow |
| Inbred line | A411 | DBM | unknow |
| Inbred line | A412 | QLM | unknow |
| Inbred line | A413 | QLF | unknow |
| Inbred line | A415 | Y22M | unknow |
| Inbred line | A416 | KM | unknow |
| Inbred line | A417 | S36F | unknow |
| Inbred line | A419 | XD29M | unknow |
| Inbred line | A420 | 12HL-1 | unknow |
| Inbred line | A421 | 12HT274 | unknow |
| Inbred line | A423 | Limin33M | unknow |
| Inbred line | A424 | DH605M | unknow |
| Inbred line | A425 | DH605F | unknow |
| Inbred line | A427 | Dayu3Fs | unknow |
| Inbred line | A428 | 702M | unknow |
| Inbred line | A429 | DH863F | unknow |
| Inbred line | A430 | 13HLD34 | unknow |
| Inbred line | A431 | Qi31922G | unknow |
| Inbred line | A432 | XD20M | unknow |
| Inbred line | A433 | XD21M | unknow |
| Inbred line | A434 | XD22M | unknow |
| Inbred line | A437 | 10CQ03 | unknow |
| Inbred line | A438 | 10CQ04 | unknow |
| Inbred line | A439 | XD26F | unknow |
| Landrace | L001 | Nong1 | Mexico |
| Landrace | L002 | Nong2 | Mexico |
| Landrace | L003 | BQYM1050 | Mexico |
| Landrace | L005 | BQYM1051 | Mexico |
| Landrace | L007 | SSHEB | Mexico |
| Landrace | L010 | XHYM | Mexico |
| Landrace | L011 | CYT | Mexico |
| Landrace | L014 | FENG261 | Mexico |
| Landrace | L015 | MYHEB | Mexico |
| Landrace | L016 | CDHYM | Mexico |
| Landrace | L019 | YJB | Mexico |
| Landrace | L020 | HEB | Mexico |
| Landrace | L022 | BQ1094 | Mexico |
| Landrace | L023 | BG1106 | Mexico |
| Landrace | L024 | XYM1181 | Mexico |
| Landrace | L026 | SSHQ1070 | Mexico |
| Landrace | L027 | BBG1124 | Mexico |
| Landrace | L028 | BH1033 | Mexico |
| Landrace | L030 | HYM1042 | Mexico |
| Landrace | L033 | BQYM | Mexico |
| Landrace | L035 | XBBG | Mexico |
| Landrace | L036 | HQ1069 | Mexico |
| Landrace | L037 | HYM1096 | Mexico |
| Landrace | L038 | EFZ1148 | Mexico |
| Landrace | L041 | ZSY1177 | Mexico |
| Landrace | L042 | HEB1072 | Mexico |
| Landrace | L044 | BDH1147 | Mexico |
| Landrace | L045 | HQ1093 | Mexico |
| Landrace | L046 | 46 | Mexico |
| Landrace | L048 | HYM1006 | Mexico |
| Landrace | L049 | LBYM1149 | Mexico |
| Landrace | L051 | HBG | Mexico |
| Landrace | L053 | BYM1131 | Mexico |
| Landrace | L054 | EYXHJ | Mexico |
| Landrace | L056 | 56 | Mexico |
| Landrace | L057 | 57 | Mexico |
| Landrace | L059 | YJB1088 | Mexico |
| Landrace | L060 | BQSZ | Mexico |
| Landrace | L063 | BYQ | Mexico |
| Landrace | L066 | WYBMY | Mexico |
| Landrace | L068 | DBBEB | Mexico |
| Landrace | L070 | BQ1025 | Mexico |
| Landrace | L072 | HMY | Mexico |
| Landrace | L073 | YLZ | Mexico |
| Landrace | L075 | BMY | Mexico |
| Landrace | L076 | JHH | Mexico |
| Landrace | L077 | HBG | Mexico |
| Landrace | L078 | XJH | Mexico |
| Landrace | L080 | XJH | Mexico |
| Landrace | L094 | GZH | Mexico |
| Landrace | L095 | BRH | Mexico |
| Landrace | L096 | PWHEB | Mexico |
| Landrace | L097 | QYM | Mexico |
| Landrace | L098 | BEB | Mexico |
| Landrace | L099 | NJZ1004 | Mexico |
| Landrace | L100 | XHJ | Mexico |
| Landrace | L102 | LHJ | Mexico |
| Landrace | L106 | 106 | Mexico |
| Landrace | L108 | 108 | Mexico |
| Landrace | L109 | 109 | Mexico |
| Landrace | L110 | 110 | Mexico |
| Landrace | L111 | 111 | Mexico |
| Landrace | L113 | 113 | Mexico |
| Landrace | L114 | 114 | Mexico |
| Landrace | L115 | 115 | Mexico |
| Landrace | L117 | 117 | Mexico |
| Landrace | L119 | 119 | Mexico |
| Landrace | L121 | 121 | Mexico |
| Landrace | L124 | 124 | Mexico |
| Landrace | L127 | 127 | Mexico |
| Landrace | L132 | 132 | Mexico |
| Teosintes | T001 | 28620 | Mexico |
| Teosintes | T002 | Guerrero | Mexico |
| Teosintes | T003 | 625 | Mexico |
| Teosintes | T004 | No. 14438 | Mexico |
| Teosintes | T005 | 27215 | Mexico |
| Teosintes | T006 | No. 13 | Mexico |
| Teosintes | T007 | CIMMYT ID: 29766 | Mexico |
| Teosintes | T008 | CIMMYT ID: 29798 | Mexico |
| Teosintes | T009 | FS1834 | Mexico |
| Teosintes | T010 | FS1841 | Mexico |
| Teosintes | T011 | FS1845 | Mexico |
| Teosintes | T012 | 2460 | Mexico |
| Teosintes | T013 | 28629 | Mexico |
| Teosintes | T014 | III.B. 10 | Mexico |
| Teosintes | T015 | Chalco | Mexico |
| Teosintes | T016 | I.A. 7 | Mexico |
| Teosintes | T017 | I.B. 29 | Mexico |
| Teosintes | T018 | BENZ 967 | Mexico |
| Teosintes | T019 | Wilkes 47259 | Mexico |
| Teosintes | T020 | FS1818 | Mexico |
| Teosintes | T021 | FS1847 | Mexico |
| Teosintes | T022 | FS1854 | Mexico |
| Teosintes | T023 | IA1 | Mexico |
| Teosintes | T024 | IA2 | Mexico |
| Teosintes | T025 | IA8 | Mexico |
| Teosintes | T026 | IA9 | Mexico |
| Teosintes | T027 | IA10 | Mexico |
| Teosintes | T028 | IA11 | Mexico |
| Teosintes | T029 | IA29 | Mexico |
| Teosintes | T030 | IA36 | Mexico |
| Teosintes | T031 | BENZ 967 | Mexico |
| Teosintes | T032 | Maiz de Pajaro | Mexico |

**Supplementary Table S2**. The result of descriptive statistics and ANOVA for phenotypic traits among 256 maize inbred lines.

| Parameters | EW | EGW | EL | ED | ERN | KNR | CD | HKW | KL | KW | KT |
| --- | --- | --- | --- | --- | --- | --- | --- | --- | --- | --- | --- |
| Mean | 77.842 | 63.997 | 12.093 | 3.902 | 13.608 | 21.282 | 2.438 | 25.492 | 9.463 | 8.054 | 5.009 |
| *SD* | 31.096 | 27.440 | 2.472 | 0.521 | 2.418 | 5.962 | 0.396 | 5.943 | 1.256 | 0.921 | 0.873 |
| Minimum | 6.020 | 3.660 | 3.769 | 1.069 | 4.000 | 5.000 | 1.075 | 10.100 | 5.507 | 4.075 | 2.578 |
| Maximum | 214.800 | 186.700 | 21.540 | 6.866 | 24.000 | 42.000 | 4.296 | 44.440 | 26.370 | 12.080 | 9.552 |
| *F* (Gen) | 19.758*** | 19.048*** | 18.804*** | 28.566*** | 23.178*** | 13.280*** | 39.587*** | 40.800*** | 21.356*** | 24.552*** | 11.708*** |
| *F* (Env) | 197.969*** | 144.906*** | 738.594*** | 73.429*** | 112.417*** | 191.522*** | 355.398*** | 2204.416*** | 131.519*** | 37.992*** | 606.182*** |
| *F* (Gen*Env) | 4.359*** | 4.276** | 3.833*** | 3.102*** | 2.490*** | 3.245*** | 3.408*** | 8.087*** | 3.553*** | 2.732*** | 3.359*** |
| *F* （Block） | 44.544*** | 40.810*** | 32.730*** | 15.470*** | 9.828** | 24.599*** | 2.012 | 8.309** | 10.331** | 5.632* | 0.036 |

*** indicates a statistical significance at *p* < 0.001 level
